# CCL18 Exhibits a Regulatory Role through Inhibition of Receptor and Glycosaminoglycan Binding

**DOI:** 10.1371/journal.pone.0072321

**Published:** 2013-08-12

**Authors:** Sonja C. Krohn, Pauline Bonvin, Amanda E. I. Proudfoot

**Affiliations:** Department of Immunology, Merck Serono Geneva Research Centre, Geneva, Switzerland; University of York, United Kingdom

## Abstract

CCL18 has been reported to be present constitutively at high levels in the circulation, and is further elevated during inflammatory diseases. Since it is a rather poor chemoattractant, we wondered if it may have a regulatory role. CCL18 has been reported to inhibit cellular recruitment mediated by CCR3, and we have shown that whilst it is a competitive functional antagonist as assessed by Schild plot analysis, it only binds to a subset of CCR3 receptor populations. We have extended this inhibitory activity to other receptors and have shown that CCL18 is able to inhibit CCR1, CCR2, CCR4 and CCR5 mediated chemotaxis, but has no effect on CCR7 and CCR9, nor the CXC receptors that we have tested. Whilst CCL18 is able to bind to CCR3, it does not bind to the other receptors that it inhibits. We therefore tested the hypothesis that it may displace glycosaminoglycan (GAG) chemokines bound either in cis- on the leukocyte, or in trans-presentation on the endothelial surface, thereby inhibiting the recruitment of leukocytes into the site of inflammation. We show that CCL18 selectivity displaces heparin bound chemokines, and that chemokines from all four chemokine sub-classes displace cell bound CCL18. We propose that CCL18 has regulatory properties inhibiting chemokine function when GAG-mediated presentation plays a role in receptor activation.

## Introduction

Chemokines (chemotactic cytokines) constitute a large family of cytokines that are so named based on their ability to recruit leukocytes. They act primarily as part of the selective movement of specific cell types into and out of specific tissue microenvironments during basal trafficking as well as inflammatory processes. Chemokines are divided into four different subfamilies (CXC or α-, CC or β-, CX _3_C or γ- and C or δ-chemokines) [1,2]. The majority of activities attributed to chemokines are induced by interaction with seven-transmembrane G protein-coupled receptors (7-TM GPCRs) expressed on their target cells. Approximately 50 chemokines and 20 chemokine receptors have been identified to date, with 7-TM GPCRs identified for all but two chemokines. The chemokine-receptor system appears to be highly promiscuous, as several chemokines are able to bind more than one receptor and several receptors bind more than one chemokine. However this overlap in chemokine binding maybe due to *in vitro* studies, whereas *in vivo* the leukocyte recruitment could be highly specific and regulated based on the temporal and spatial distribution of chemokines.

Chemokines have been shown to bind to GAGs present on the surface of endothelial and leukocyte cells and the extracellular matrix [3,4]. This chemokine-GAG interaction is thought to facilitate the immobilization of chemokines resulting in the formation of localized gradients, which are required for the directional cell migration. Furthermore it was shown that the chemokine immobilization on GAGs can enable certain chemokines to oligomerize, which was shown to be essential for their *in vivo* activities [5]. GAG binding has also been proposed to play a role in receptor activation by chemokine binding to GAGs on the leukocyte surface where they can then facilitate receptor binding, defined as cis-presentation [6,7].

CCL18 was discovered by independent groups 15 years ago and was originally termed pulmonary and activation-regulated chemokine (PARC) [8], macrophage inflammatory protein-4 (MIP-4) [9,10], dendritic cell-chemokine 1 (DC-CK1) [11] and alternative macrophage activation-associated CC-chemokine-1 (AMAC-1) [12]. CCL18 has been described to induce activation of intracellular calcium mobilization [13,14] and actin polymerization [13,15], and mediate various biological functions such as chemotactic responses [8,11,13,15–20], stimulation of collagen production in fibroblasts [21,22], monocyte maturation into an M2 phenotype [23] and the generation of adaptive regulatory T cells [24]. The chemotactic response has been shown to be pertussis toxin sensitive indicating that its receptor is a member of the GPCR superfamily, but its identification has remained elusive to date.

CCL18 is constitutively present in the circulation at rather high concentrations and enhanced levels have been demonstrated in several diseases [25,26]. Therefore CCL18 might be implicated in homeostatic processes but may also play a role in several human diseases, which have been reported to be accompanied with elevated levels of CCL18, including various malignancies, fibrotic lung diseases and inflammatory joint and skin diseases [25]. Interactions of CCL18 with the chemokine receptor CCR3 have been reported, on which it exhibits antagonistic activity, but does not signal [27]. More recently an additional modulatory activity of CCL18 has been reported with the chemokine-like receptor, G protein-coupled receptor 30 (GPR30) [28], which was shown to result in the diminution of CXCR4-dependent responses. Whilst the classical 7-TM receptor for CCL18 remains to be identified, PITPNM3 has been reported to mediate the CCL18 induced recruitment of tumor cells [29].

We report here a potential anti-inflammatory role of CCL18. We extended the reported observation that CCL18 inhibits CCL11- and CCL13- induced cellular recruitment of human eosinophils mediated by CCR3 [27] and showed that it also inhibits the chemotactic responses of other CCR3 agonists, namely CCL5, CCL15 and CCL26. By studying its molecular mechanism of action on CCR3 we showed that CCL18 behaves as a competitive antagonist in functional assays. The binding capacity of radiolabelled CCL18 suggests that it is only able to bind to a sub-population of the total CCR3 receptors, which is reflected by the inefficient displacement of CCR3 bound CCL11 by CCL18. Importantly we showed that CCL18 is able to inhibit the cellular recruitment mediated by other chemokine receptors. CCL18 is able to inhibit CCR1, CCR2, CCR4 and CCR5 mediated chemotactic responses to a variety of ligands, whereas no inhibitory activity was observed on CCR7 and CCR9 mediated responses as well as the CXC receptors, CXCR3 and CXCR5. This inhibitory effect of CCL18 on the cellular recruitment of several receptors appears not to be mediated by direct binding to the receptors, but through a GAG binding mechanism since abrogation of GAG binding abrogates the inhibition. We further demonstrated that the CCL18/GAG interaction could be involved in a regulatory role of CCL18 through selectivity in the displacement of GAG bound chemokines by CCL18, suggesting a modulatory role of this interaction, both in cis- and trans-presentation.

## Methods

### Ethics statement

Blood samples of healthy donors were obtained from the “Centre de transfusion sanguine, Hôpital Universitaire de Genève, 6, rue Gabrielle Perret-Gentil, 1211 Genève 14” (http://labos.hug-ge.ch/) and analysed anonymously. In accordance with the ethical committee of the Geneva Hospital, the blood bank obtained informed consent from the donors, who are thus informed that part of their blood will be used for research purposes.

### Reagents

Chemokines were purchased from PeptroTech or produced as previously described [30]. Radiolabelled CCL11, ^125^I-CCL11 (catalogue number NEX314) was purchased from PerkinElmer.^^125^^ I-CCL18 was obtained from Anawa. The heparin used in the assay was obtained from Sigma (unfractionated heparin sodium salt, 5-30 kDa, catalogue number H3393).

### Inhibition of Chemotaxis

Inhibition of chemotaxis was performed using 96-well microplates (Neuro Probe ChemoTx) with 300.19/CCR1 and L1.2/CCR2-5 transfectants. L1.2 transfectants were cultured in RPMI 1640 medium containing 10% inactivated fetal calf serum (FCS), L-glutamine (5 mM), 50 U/ml Penicillin/Streptomycin (P/S), 0.05 mM β-mercaptoethanol and 0.6 mg/ml Geneticin G-418. 300.19 transfectants were cultured in RPMI 1640 medium containing 10% inactivated FCS, Glutamax (1 mM), 50 U/ml P/S, 0.05 mM β-mercaptoethanol, 1 mM Minimum essential medium nonessential amino acid (MEN- NEAA), 1 mM Sodium Pyruvate, 1.5 µM Puromycin. L1.2 and 300.19 transfectants were maintained in a humidified incubator at 37 °C with 5% CO_2_ and diluted every three days to a final concentration of 0.5×10^6^ cells/ml. The day before the inhibition assay, n-butyric acid (5 mM) was added to the culture medium. At the day of the experiment cells were centrifuged for 5 min at 600 × *g* and suspended at a concentration of 3×10^6^ cells/ml in RPMI medium without red phenol containing 5% FCS, 5 mM L-glutamine and 50 U/ml P/S. Inhibition of chemotaxis was performed using:

1 A constant concentration of the receptor agonist close to the value corresponding to 80% of the maximal effective concentration (EC_80_) incubated with a serial dilution of the antagonist covering the range from 10^-6^-10^-12^ M in RPMI medium without red phenol containing 5% FCS, 5 mM L-glutamine and 50 U/ml P/S. Agonist and antagonist were placed in the lower chamber of the chemotaxis plate (33 µl) and a membrane of 8 µm pore size was placed on the plate. 20 µl of cells (60×10^4^ cells) were placed on top of the membrane of each well.
2 A dose response of the receptor agonist in the presence of a constant concentration of receptor antagonist. The agonist was diluted at 0.01 mg/ml (1.25 µM) in RPMI medium without red phenol containing 5% FCS, 5 mM L-glutamine and 50 U/ml P/S containing 0 nM, 100 nM, 1 µM, 10 µM or 40 µM CCL18 and serially diluted 4-fold to cover a concentration range from 10^-6^-10^-12^ M. Agonist and antagonist were placed in the lower chamber (33 µl) of the chemotaxis plate, followed by a membrane of 8 µm pore size and 20 µl of cells (60×10^4^ cells).

The chambers containing the L1.2 transfectants were incubated for 4 h and the 300.19 transfectants for 2 h at 37 °C with 5% CO_2_. The cells were removed with PBS and migrated cells were transferred to a black 96-well plate by using a 96-well funnel adaptor. Cells were frozen overnight at -80 °C and the number of migrated cells was determined using the Cell Proliferation assay CyQUANT kit. The fluorescence bound to DNA after cell lysis was measured at 480 nm excitation and 520 nm emission using a Fluorimeter reader.

### Ca^2+^ mobilization Assay

The Ca^2+^ mobilization assay was performed using L1.2/CCR3 transfectants as previously described [31]. The day before the inhibition assay, n-butyric acid (5 mM) was added to the culture medium. Cells were added at a concentration of 10,000 cells per well. CCL11 was dissolved at 4 µM and diluted by 2-fold serial dilutions to cover a range from 10^-10^ to 10^-6^ M. For Schild analysis, assays were performed with serial dilutions of CCL11 in the presence of CCL18 at a final concentration of 10 nM, 100 nM, 500 nM, 1 µM, 5 µM or 10 µM CCL18.

### Schild Plot Analysis of Ca^2+^ Mobilization Assay

EC_50_-values were calculated using the Graph Pad Prism 5 software. EC_50_ dose ratios were calculated by the division of the EC_50_ values of the maximal response of CCL11 induced Ca^2+^ mobilization in the presence of a specific antagonistic concentration by the EC_50_ value without antagonist. Schild plot analysis were performed with log [dose ratio -1] as the ordinate and log [molar concentration of the antagonist] as the abscissa to estimate the slope and pA_2_ value.

### Equilibrium Competition Binding Assays

Receptor binding assays were carried out on primary cells or chemokine receptor transfectants as previously described using the MultiScreen HTS 96-well filtration system [32]. Briefly the assay was performed using iodinated chemokine at a final concentration of 0.1 nM and 0.1 ×10^6^ cells/well. The competitor was diluted serially to cover a range between 10^-6^ to 10^-12^ M unless stated otherwise. The plates were incubated at room temperature under gentle agitation for 4 h. The filter plates were washed three times using a vacuum pump in order to remove unbound iodinated chemokine. Finally scintillant was added to each well and the radioactivity measured using a β-scintillation counter**.**

### Receptor binding by Flow cytometry

L1.2 transfectants were cultured and activated as described above. On the day of experiment, cells were suspended at 5 ×10^6^ cells/ml in FACS buffer (PBS containing 0.1% bovine serum albumin and 0.01% azide). Alexa 647-CCL18 (Almac) was added to 10^6^ cells at a final concentration of 1, 10, or 100 nM and cells were incubated for 1 h at 4 °C in the dark. Cells were then centrifuged and washed twice with FACS buffer before being passed through a FACSCalibur flow cytometer (BD Biosciences). Data were analysed with CellQuest Pro software (BD Biosciences).

### Generation of ^44^AAGA^47^-CCL18

^44^AAGA^47^-CCL18 was generated and produced as previously described [33]. Briefly the ^44^AAGA^47^-CCL18 mutant was generated by PCR mutagenesis using the primer pairs ^44^AAGA^47^ _forward 5`CCT CCT AAC CGC CGC CGG CGC CCA GAT CTG TGC TGA CCC C and ^44^AAGA^47^ _reverse 5` GGG GTC AGC ACA GAT CTG GGC GCC GGC GGC GGT TAG GAG G. The mutations were introduced in a single step PCR reaction followed by digesting the parental plasmid DNA with *Dpn1*. The protein was produced by transient expression carried out in HEK293-6E cells and purified by Ni^2+^ affinity chromatography.

### Purification of peripheral blood leukocytes (PBL)

Mononuclear cells were purified from fresh blood by Ficoll gradient centrifugation (Ficoll-Paque PLUS, GE Healthcare). The mononuclear fraction was separated further into monocytes and lymphocytes by allowing the monocytes to adhere to tissue culture plastic in RPMI 1640 containing 10% FCS, 5 mM L-Glutamine and 50 U/ml Penicillin/Streptomycin (P/S) for 120 min at 37 °C. The nonadherent lymphocytes in the supernatant were removed and used for bioassays.

### Immobilized Heparin Competition Binding Assay

Heparin binding assays were performed by incubating 1 µM chemokines on heparin-coated plates for 30 min. Heparin-bound chemokines were competed by adding 5 M NaCl, 1 µM, 100 nM or 10 nM CCL18 and incubated for 4 h at room temperature under gentle agitation. Supernatant was used to determine the amount of displaced chemokine using the Luminex technology (Custom Human 27-Plex Cytokine Panel, LEGENDplex).

### Data plotting and statistical analysis

Graphical representation was performed using GraphPad Prism version 6.0. To evaluate statistical significance t-test analysis was applied. Statistical significance was assigned as follows: P > 0.05 ns; P < 0.05*; P < 0.01**; P < 0.001***.

## Results

### CCL18 is a competitive antagonist of CCR3

**Figure 1:**
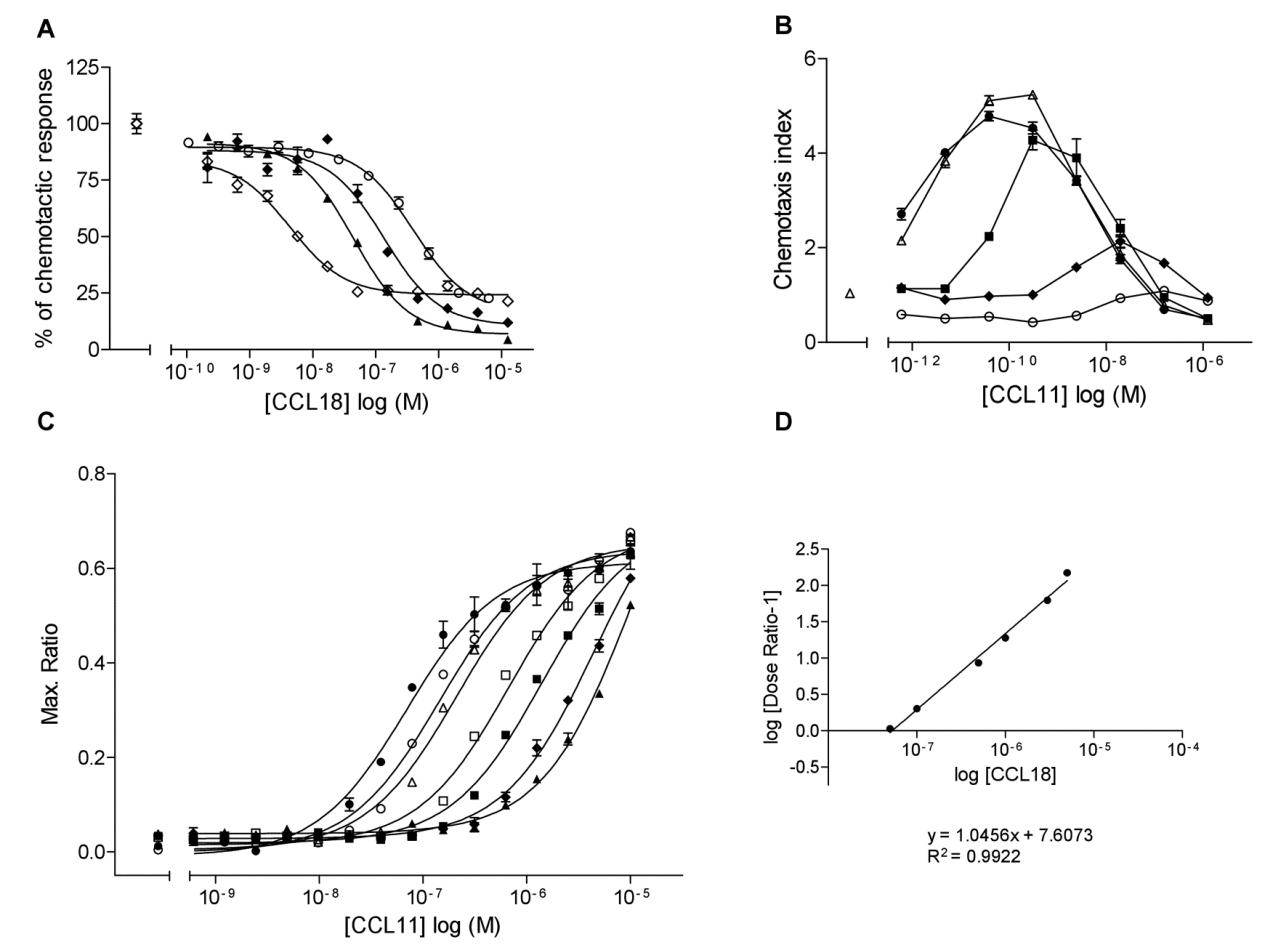
CCL18 inhibits chemotactic responses induced by several CCR3 agonists and behaves as a competitive antagonist in functional studies. A) Inhibition of chemotaxis assay using L1.2/CCR3 transfectants. Chemotaxis inhibition assays were performed using a constant concentration of receptor agonist close to the EC_80_, 1 nM CCL11 (○), 10 nM CCL5 (▲), 100 nM CCL15 (◊) and 10 nM CCL26 (♦) in the presence of an increasing concentration of CCL18. CCL18 inhibits the chemotactic responses induced by CCL5 (IC_50_: 40.79 nM), CCL11 (IC_50_: 396.7 nM), CCL15 (IC_50_: 2.86 nM) and CCL26 (IC_50_: 123.4 nM). Data are expressed in % of chemotactic response. Data points are in triplicate. The number of experiments is indicated in Table 1. B) CCL18 inhibits CCL11-induced chemotactic responses of L1.2/CCR3 transfectants. Inhibition of chemotaxis was performed using a dose–response of CCL11 (●) in the presence of a constant concentration of 100 nM (Δ), 1 µM (■), 10 µM (♦) and 40 µM (○) CCL18. Data points are in triplicate. Data are expressed as mean ± SEM. One representative experiment out of two is shown. C) Ca^2+^ mobilization assay in L1.2/CCR3 transfectants after stimulation with a dose response of CCL11 (●) in the presence of a constant concentration of 50 nM (○), 100 nM (Δ), 500 nM (□), 1 µM (■), 3 µM (♦) and 5 µM CCL18 (▲). The presence of CCL18 resulted in an increase the EC_50_ of the CCL11-induced Ca^2+^ release in a concentration dependent manner: 0 nM CCL18 (EC_50_: 69.93 nM), 50 nM CCL18 (EC_50_: 144.4 nM), 100 nM CCL18 (EC_50_: 210.6 nM), 500 nM CCL18 (EC_50_: 669.5 nM), 1 µM CCL18 (EC_50_: 1395 nM), 3 µM CCL18 (EC_50_: 4439 nM), 5 µM CCL18 (EC_50_: 10530 nM). Data points are in duplicate. One representative experiment out of two is shown. D) Investigation of the effect of CCL18 on CCR3-mediated cellular responses by Schild regression analysis. The EC_50_ values obtained in Ca^2+^ mobilization studies were used to calculate dose ratios. The dose ratios obtained were plotted as a regression of log (dose ratio -1) versus log of molar concentrations of the antagonist CCL18. The results revealed that CCL18 is compatible with a competitive type of antagonism. One representative experiment out of two is shown.

The ability of CCL18 to inhibit CCL11- and CCL13-induced chemotactic responses of human eosinophils has previously been reported [27]. We show here that it is able to inhibit the chemotactic responses of other CCR3 agonists (Figure 1A). Using EC_80_ concentrations, 1 nM CCL11, 10 nM CCL5 and CCL26 and 100 nM CCL15, the IC_50_ values were 317.6 ± 48.77 nM for CCL11, 40.79 nM for CCL5, 2.86 nM for CCL15 and 123.4 nM for CCL26. Thus CCL18 inhibits CCR3 mediated chemotactic responses induced by several CCR3 agonists.

We next investigated the molecular mechanism of inhibition to determine whether CCL18 acts as an allosteric modulator or a competitive antagonist. Chemotactic responses to a dose response of CCL11 in the presence of an increasing constant concentration of CCL18, showed a dextral shift in the presence of 1, 10 and 40 µM (Figure 1B). However the maximal response to the CCL11 induced chemotaxis decreased concomitantly, preventing a Schild plot analysis.

Calcium mobilization assays were therefore performed using L1.2/CCR3 transfectants. CCL11 induced a concentration-dependent mobilization of calcium release with an EC_50_ of 54.53 ± 15.4 nM. Inhibition of CCL11 induced calcium mobilization was performed with incremental constant concentrations of CCL18. Again, dextral shifts in the responses were observed, which reached the plateau maximum (Figure 1C). The EC_50_ values obtained were used for subsequent Schild plot analysis [34], which refer to an equation in which the EC_50_ concentrations of agonist are used to calculate dose ratios. These ratios refer to equiactive concentrations of agonist measured in the presence and absence of antagonist. The dose ratios obtained were plotted as a regression of log (dose ratio -1) versus log of molar concentrations of the antagonist CCL18. The regression of log [Dose Ratio -1] versus log [CCL18] gave a linear slope of 0.994 ± 0.052, which defines a competitive mode of antagonism (Figure 1D).

The linear relationship enables the graphical estimation of the antagonistic potency, which can be estimated by calculating the pA_2_ value [34]. The pA_2_ value of a competitive antagonist is defined as the negative logarithm of the molar concentration of an antagonist that equals to a log (Dose Ratio -1) of 0, which reduces the effect of a dose of agonist to that of half of the agonistic dose [35]. Thus the pA_2_ value can be equalized to the pK_B_ value in a linear Schild regression, which refers to the affinity of the antagonist for the receptor. Based on the Schild regression analysis pA_2_ values of -7.34 and -7.28 were obtained, in two independent experiments. This results in an antagonistic affinity of 45.7 nM and 52.48 nM of CCL18 on CCR3.

### Equilibrium competition CCR3 receptor binding assays

**Figure 2:**
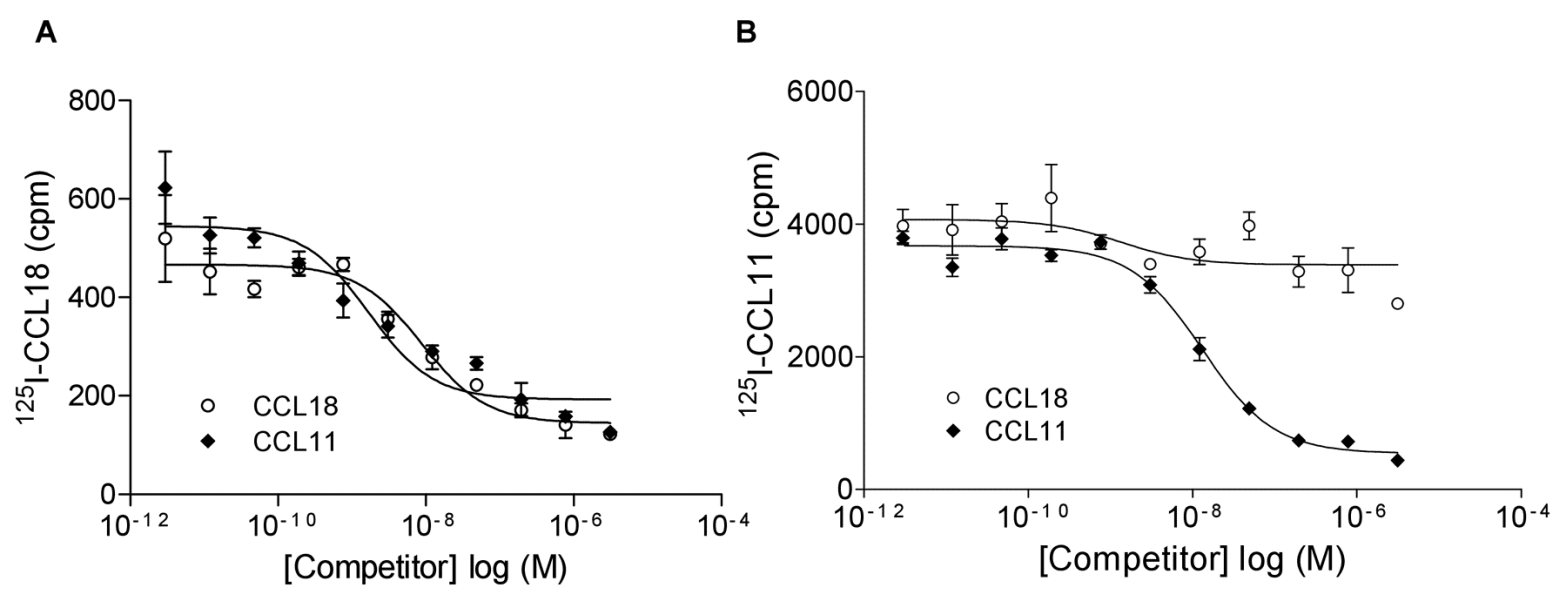
CCL18 does not compete CCL11 for CCR3 binding. Equilibrium competition binding assay using L1.2/CCR3 transfectants. A) Binding of ^125^I-CCL18 was competed by an increasing concentration of CCL18 (IC_50_: 8.86 nM) and CCL11 (IC_50_: 1.69 nM). One representative experiment out of four is shown. B) ^125^I-CCL11 binding was competed by an increasing concentration of CCL11 (IC_50_: 13.2 nM), whereas only a partial displacement was obtained in the presence of CCL18. One representative experiment out of two is shown. Data points are in triplicate and are expressed as mean ± SEM.

Competition for ^125^I-CCL18 or ^125^I-CCL11 binding to L1.2/CCR3 transfectants did not show similar heterologous displacement. Both CCL18 and CCL11 competed for ^125^I-CCL18 binding with IC_50_ values of 6.80 ± 1.39 nM and 21.91 ± 15.92 nM, respectively (Figure 2A). However intriguing results were obtained by examining the ability of CCL18 and CCL11 to displace ^125^I-CCL11. CCL11 competed ^125^I-CCL11 binding with an IC_50_ of 7.4 ± 3.78 nM, whereas only a minor displacement by CCL18 was obtained (Figure 2B). However the maximum number of counts of ^125^I-CCL18 bound was approximately 500 counts per minute (cpm) compared to 4000 cpm for ^125^I-CCL11, indicating a lower binding capacity of CCL18 to CCR3. The radioactivity of the iodinated proteins was between 2000 and 2200 Ci/mmol and their identical specific radioactivity was adjusted and verified. Therefore the displacement of ^125^I-CCL11 by CCL18 observed corresponds to approximately 10% of the bound tracer.

### CCL18 antagonizes signalling mediated by CCR1, CCR2, CCR4 and CCR5

**Figure 3:**
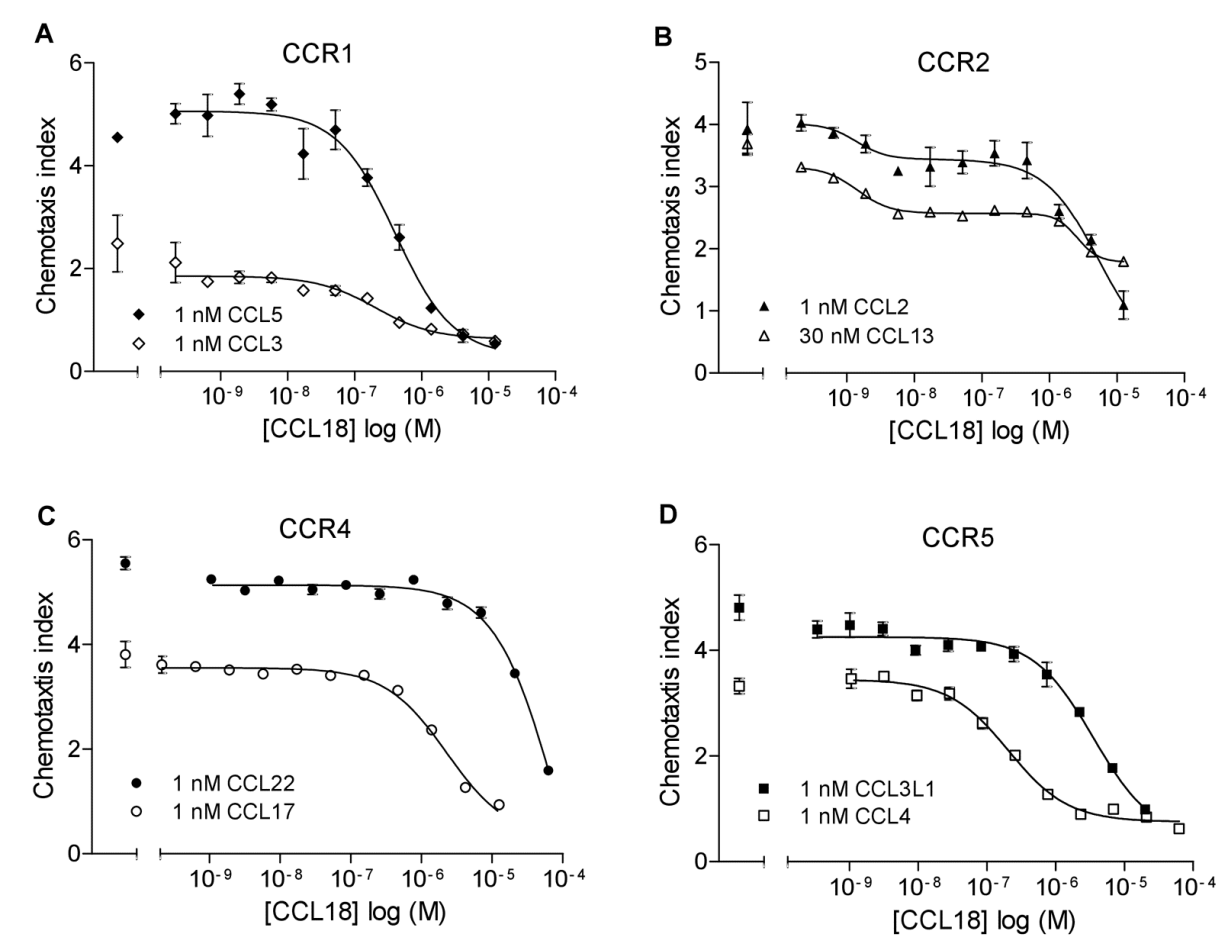
CCL18 inhibits CCR1, CCR2, CCR4 and CCR5-mediated chemotactic responses. A) Inhibition of 1 nM CCL5 (IC_50_: 403.3 nM) and 1 nM CCL3 (IC_50_: 263.1 nM) mediated chemotactic responses of 300.19/CCR1 transfectants. B) Inhibition of 1 nM CCL2 (IC_50_: 1.38 nM and 5.3 µM) and 30 nM CCL13 (IC_50_: 1.43 nM and 2.58 µM) mediated chemotactic responses of L1.2/CCR2 transfectants. C) Inhibition of 1 nM CCL22 (IC_50_: 85.5 µM) and 1 nM CCL17 (IC_50_: 2.28 µM) mediated chemotactic responses of L1.2/CCR4 transfectants. D) Inhibition of 1 nM CCL3L1 (IC_50_: 3.52 µM) and 1 nM CCL4 (IC_50_: 205.4 nM) mediated chemotactic responses of L1.2/CCR5 transfectants. The number of experiments is indicated in Table 1. Data are expressed as mean ± SEM.

**Figure 4:**
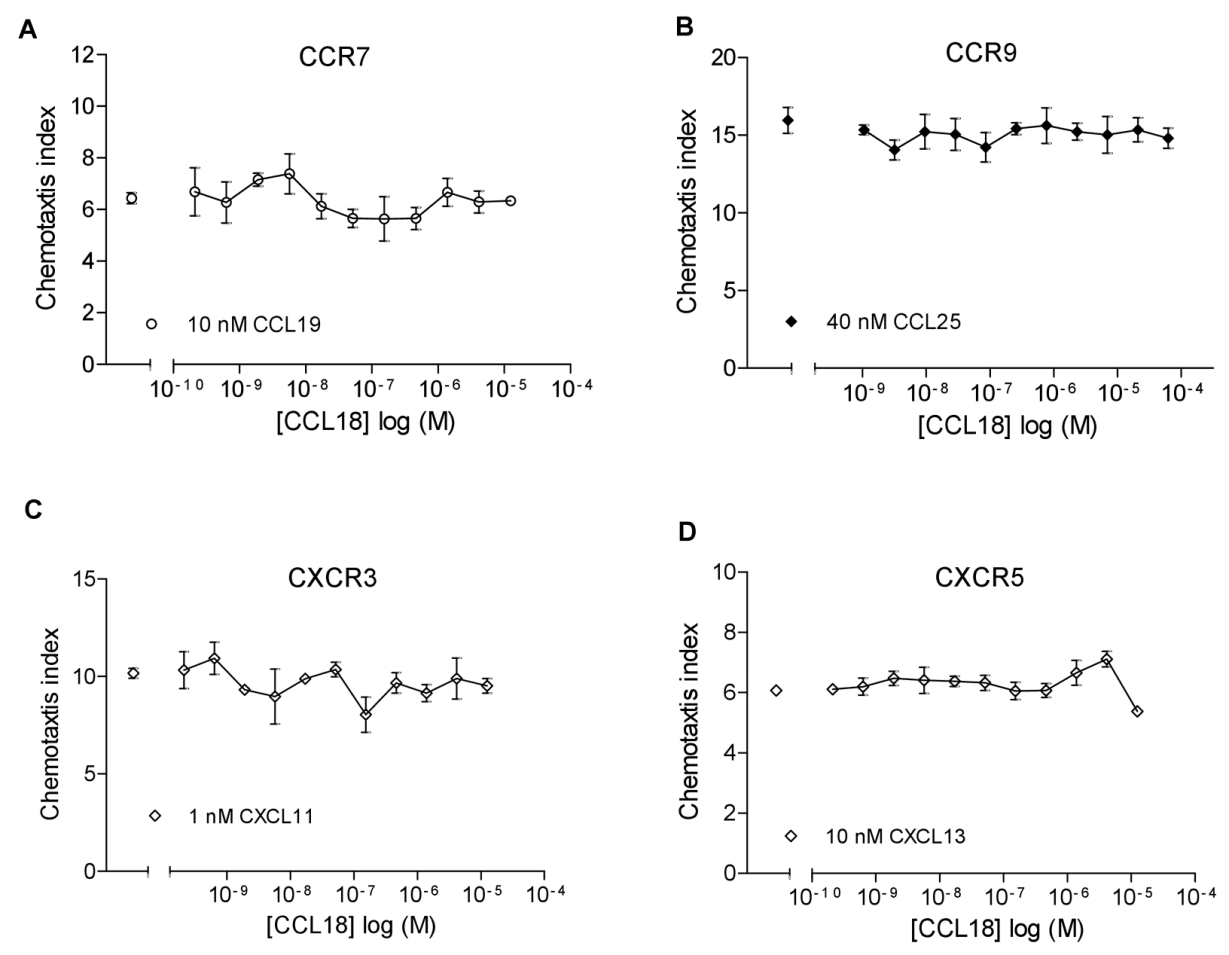
CCL18 does not have an inhibitory effect on CCR7, 9, CXCR3 and 5-mediated chemotactic responses. Inhibition of chemotaxis assay of A) 10 nM CCL19 mediated chemotactic response of T lymphocytes. One representative experiment out of two is shown. B) 40 nM CCL25 mediated chemotactic response of MOLT-4. One representative experiment out of four is shown. C) 1 nM CXCL11 mediated chemotactic response of L1.2/CXCR3 transfectants and D) 10 nM CXCL13 mediated chemotactic response of L1.2/CXCR5 transfectants in the presence of an increasing concentration of CCL18. One representative experiment out of two is shown. Data are expressed as chemotaxis index ± SEM. Data points are in triplicate.

To extend the observation that CCL18 inhibits CCR3-mediated responses we investigated its inhibitory effect further on CC- and CXC-mediated responses in chemotaxis inhibition studies. We analysed the inhibitory effect of CCL18 on CCR1, 2, 4, 5, 7 and 9 as well as CXCR3 and 5 mediated responses using EC_80_ ligand concentrations of the agonists. As shown in Figure 3, CCL18 inhibited ligand induced chemotaxis mediated by CCR1, CCR2, CCR4 and CCR5, and the results are summarized in Table 1. It should be noted that the IC_50_ values show potencies that are reminiscent of chemokine-GAG interactions as opposed to nanomolar values usually observed for receptor interactions. Simple inhibition was observed with the exception of CCR2, where a two site inhibition was systematically observed. However, no inhibitory effect was observed on CCR7, 9 and CXCR3 and 5 mediated responses (Figure 4).

**Table 1:** CCL18 inhibition of chemotactic responses.

**Receptor**	**Agonist**	**Conentration in [nM]**	**IC_50_ in [nM]**	**n**
CCR1	CCL3	1	825.7 ± 351.3	3
	CCL5	1	803.2 ± 321.8	3
CCR2	CCL2	1	1.6 ± 0.3	3
			4100 ± 1400	
	CCL13	30	10.5 ± 8.5	4
			2700 ± 900	
CCR3	CCL5	10	40.8	1
	CCL11	1	317.6 ± 48.8	7
	CCL15	100	2.9	1
	CCL26	10	123.4	1
CCR4	CCL17	1	1500 ± 600	2
	CCL22	1	42600 ± 20900	3
CCR5	CCL3L1	1	6000 ± 2900	2
	CCL4	1	213.0	1
	CCL4	10	6900 ± 4900	2
	CCL5	1	13600 ± 10000	2

IC_50_ values are expressed as mean ± SEM except for results from a single experiment.

### Binding of Alexa 647-CCL18 to receptor transfectants

**Figure 5:**
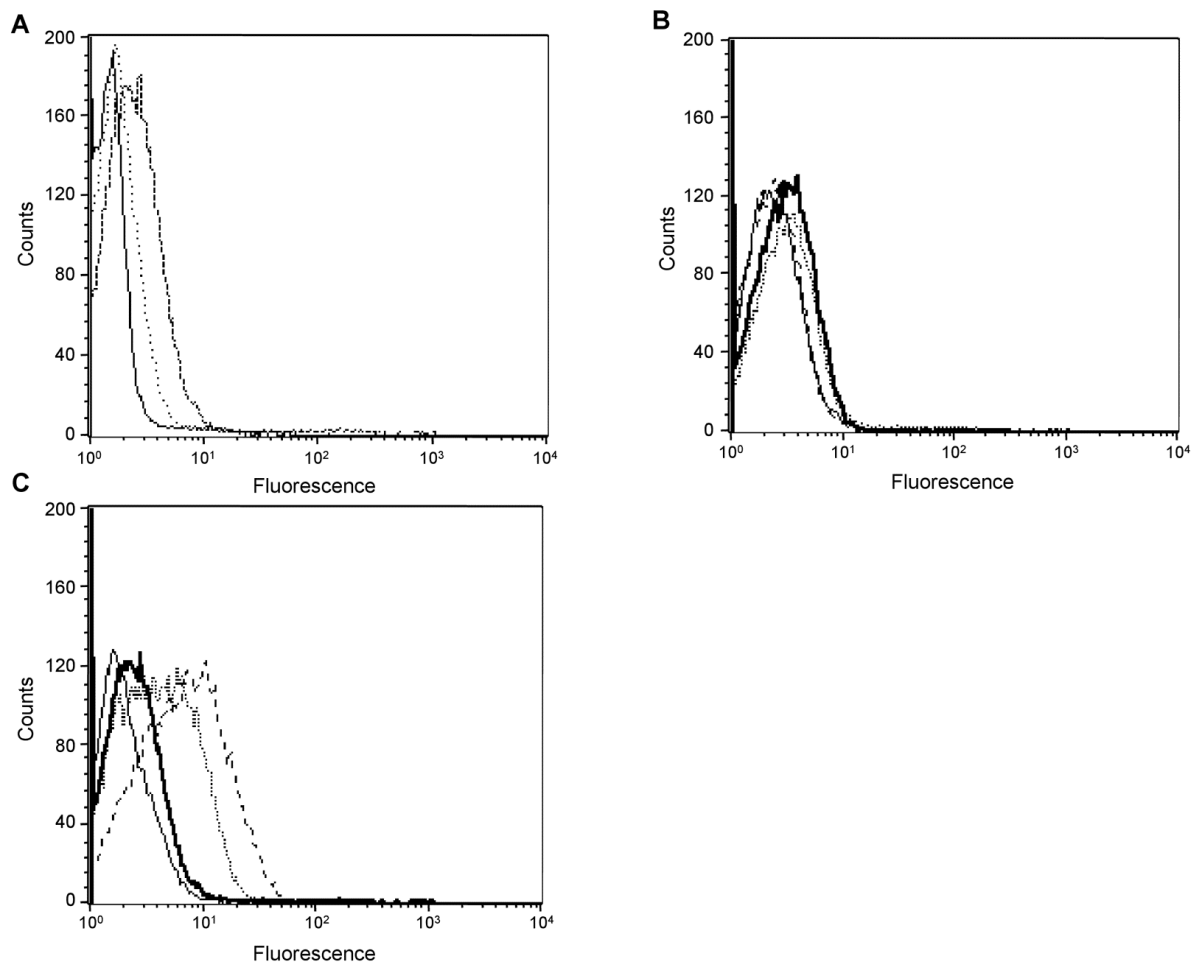
Binding of CCL18 to L1.2 receptor transfectants. A) Untransfected L1.2 cells were incubated with 1 (―), 10 (····) or 100 (-‒) nM Alexa 647-CCL18 and analyzed by flow cytometry. B) Incubation of 100 nM Alexa 647-CCL18 with L1.2/CCR2 (····), CCR4 (-‒) or CCR5 (▬) cells and untransfected L1.2 (―) as negative control. C) A dose-dependent binding of CCL18 to L1.2/CCR3 transfectants was clearly visible when using 1 (―), 10 (····) or 100 (-‒) nM Alexa 647-CCL18. Untransfected L1.2 stained with 100 nM CCL18 (▬) are shown as control. One representative experiment out of two is shown.

FACS analysis of the ability of Alexa 647-CCL18 binding to the L1.2 parental cell line and to CCR2, CCR3, CCR4 and CCR5 L1.2 transfectants demonstrated that specific binding was only observed in the case of CCR3 (Figure 5). The high propensity of CCL18 to bind to cell surface GAGs is manifest in that CCL18 demonstrates low binding to untransfected cells, which is again seen with the CCR2, CCR4 and CCR5 transfectants, but only in the case of the CCR3 transfectants is specific dose related binding observed. Thus the inhibition of cell migration seen in Figure 4 cannot be due to competition for receptor binding in the case of CCR2, CCR4 and CCR5, leading to the hypothesis that GAG binding may be playing a role.

### The BBXB motif in the 40’s loop of CCL18 contributes to receptor inhibition

**Figure 6:**
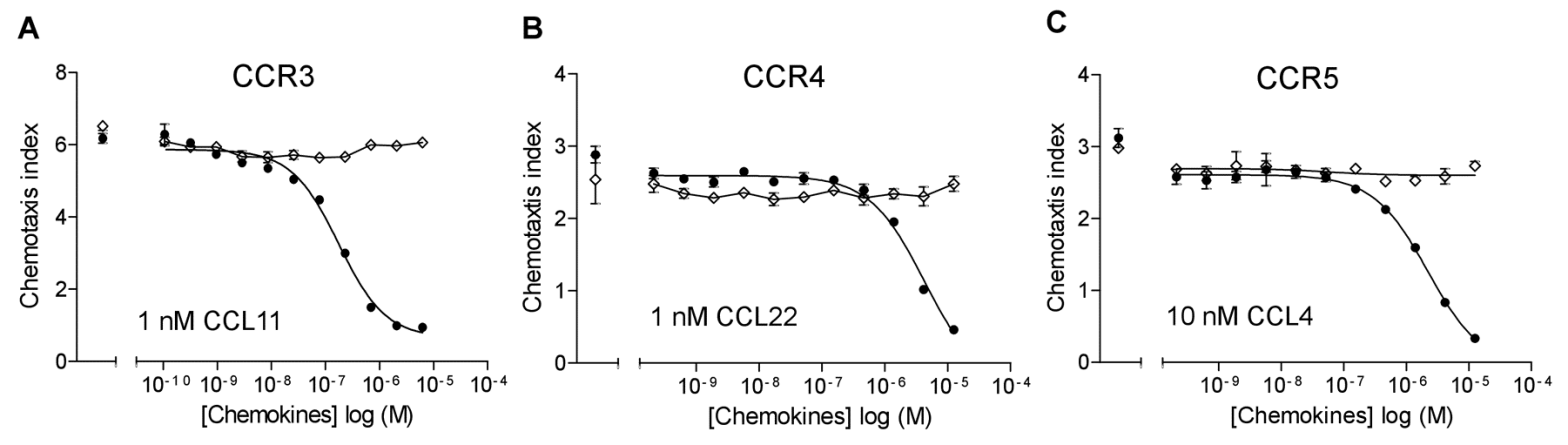
The ^44^KRGR^47^ cluster of CCL18 in the 40’s loop is involved in the interaction with CCR3, CCR4 and CCR5. A) L1.2/CCR3 transfectants migrating to 1 nM CCL11 was inhibited by CCL18-6His (●) (IC_50_: 168.4 nM). B) L1.2/CCR4 transfectants migrating to 1 nM CCL17 was inhibited in the presence of CCL18-6His (IC_50_: 4.24 µM). C) CCL18-6His inhibited migration of L1.2/CCR5 transfectants induced by 10 nM CCL4 with an IC_50s_ of 2.2 µM. No inhibitory effect was obtained in the presence of ^44^AAGA^47^-CCL18-6His (◊). One representative experiment out of two is shown.

This BBXB motif, located in the 40’s loop, has been shown to be responsible for the GAG binding ability of CCL18 [33]. The ability of the GAG binding mutant ^44^AAGA^47^-CCL18, in which the basic residues of the ^44^KRGR^47^ cluster are mutated into alanine residues, to bind to CCR3 in receptor binding studies was assessed. The ability of ^44^AAGA^47^-CCL18 to compete for ^125^I-CCL18 binding was impaired compared to CCL18 (data not shown), indicating a role of the ^44^KRGR^47^ motif in CCR3 receptor binding and in inhibition of functional responses (Figure 6A). Similarly ^44^AAGA^47^-CCL18 was unable to inhibit CCR4 and CCR5 mediated chemotaxis (Figure 6B and C).

**Figure 7:**
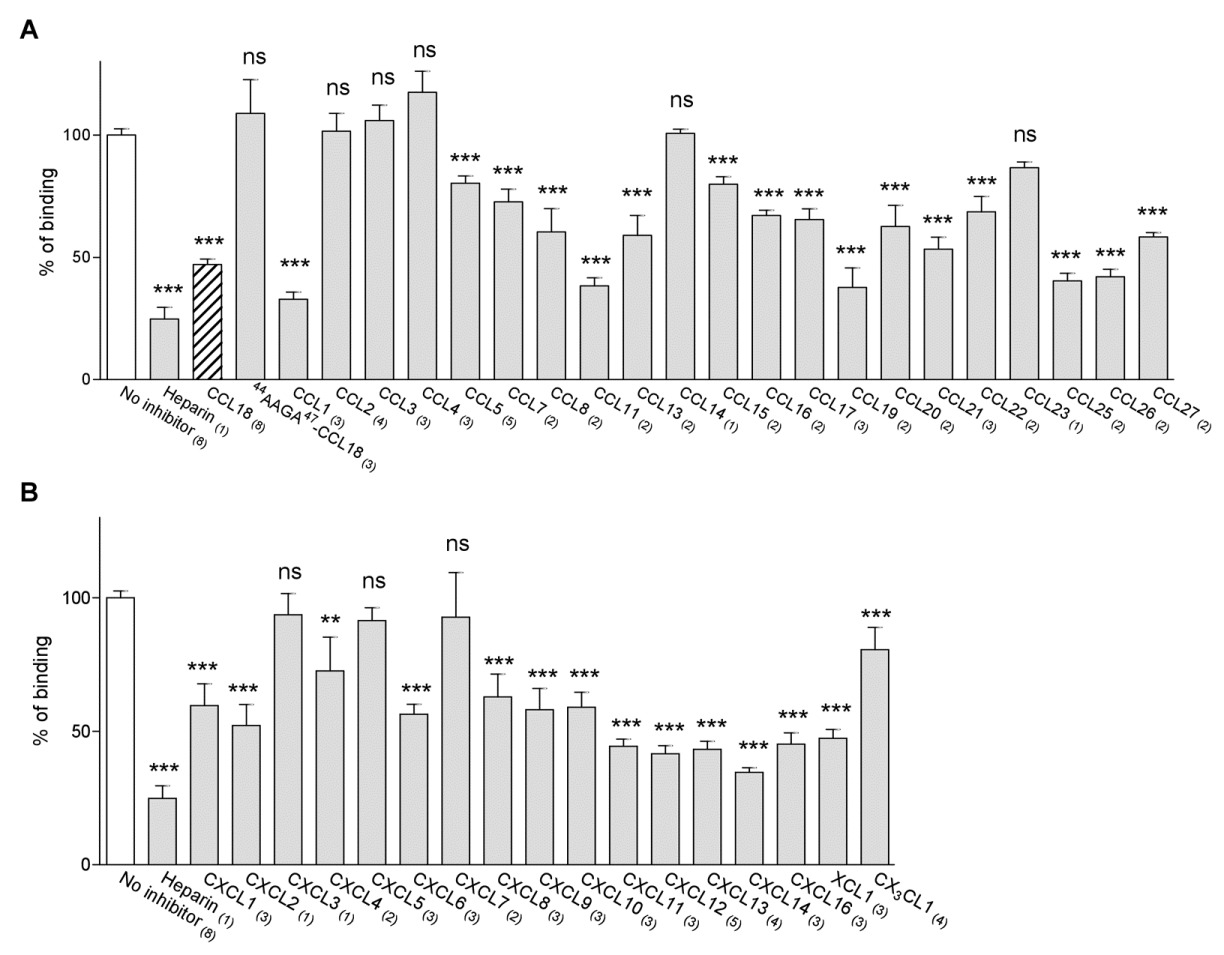
A broad spectrum of chemokines displaces ^125^I-CCL18 bound to the cell surface of PBL. Equilibrium competition binding assay on PBL using ^125^I-CCL18 and a full spectrum of chemokines. A) CC-chemokines and B) CXC-chemokines, XC and CX _3_C chemokines. Binding of ^125^I-CCL18 was competed by a constant concentration of 1 µM competitor. The binding of ^125^I-CCL18 in the absence of competitor was equalized to 100% of binding (No inhibitor). The data are expressed in % of total binding. Graph represents the mean ± SEM of *n* independent experiments (in brackets) performed in triplicate.

### Heterologous displacement of immobilized chemokines

The ability of chemokines to displace radiolabelled CCL18 bound to PBLs was assessed. As shown in Figure 7, displacement was only observed with certain chemokines but belonging to both major sub-classes. CCL1, 11, 19, 25 and 26 were almost as efficacious as heparin, whilst intermediate displacement was observed for other CCR2, 3, 4 and 5 ligands. In accordance with their weak GAG binding properties, CCL3 and CCL4 showed no displacement, but CCL2 and CXCL8, which have similar GAG binding properties [36] differed in this assay as CCL2 was unable to displace CCL18, whereas CXCL8 was efficacious. CCL5 which has been shown to have very strong binding capacity to GAGs, and shows a wide range of selectivity, with three orders of magnitude differences in IC_50_ values for various GAG families [36], was unable to achieve complete displacement of CCL18. In general the CXC ligands were more efficient in displacing CCL18 bound to PBLs.

**Figure 8:**
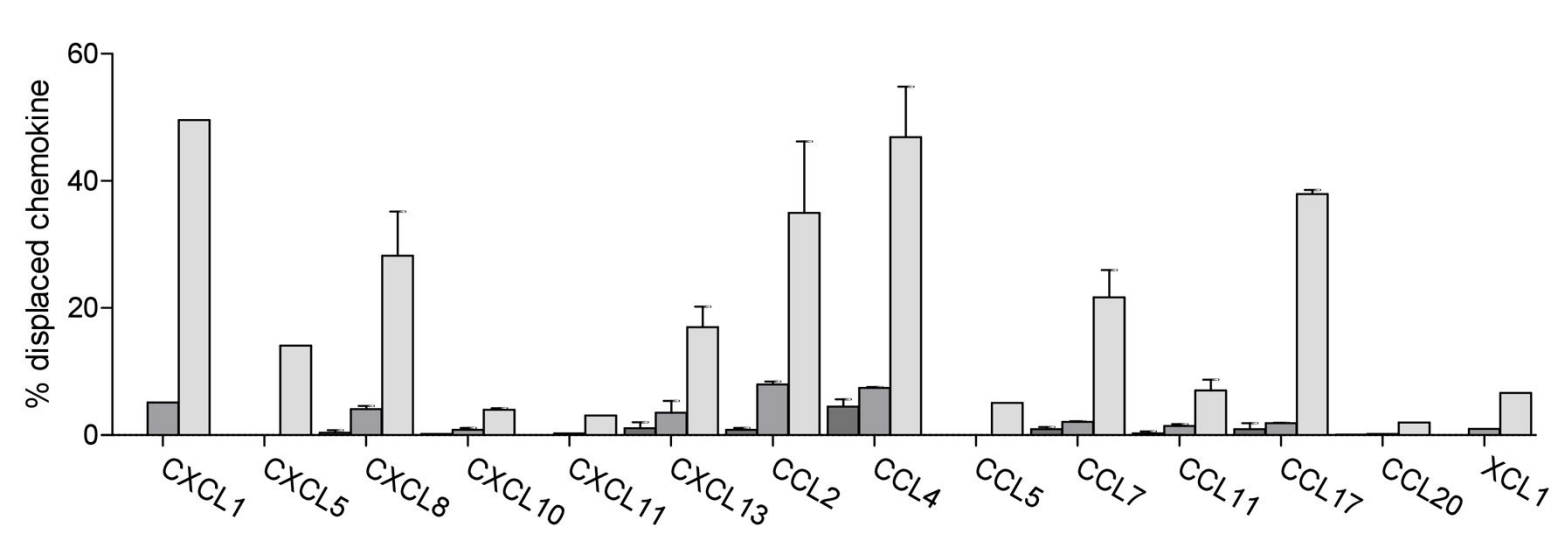
CCL18 displacement of heparin bound chemokines. Heparin bound chemokines were displaced by 10 nM (dark gray), 100 nM (gray) and 1 µM (light gray) CCL18. Chemokines in the supernatants were detected using the Luminex technology. Amount of chemokine displaced by 5 M NaCl is set to 100% and data are expressed as % of displaced chemokine. Graph represents the mean ± SEM. Data for CXCL8, 10, 13, CCL2, 4, 7, 11 and 17 represent two independent experiments. Using 1 µM CCL18 as competitor a displacement of up to 50% GAG-bound chemokine was obtained.

We then performed the analysis in the opposing sense where we investigated the ability of CCL18 to displace chemokines bound to heparin, which is an *in vitro* approximation of trans-presentation. The analysis was limited to the chemokines that were available in the Luminex kit. We determined the total amount of chemokine bound to heparin, which varies among different chemokines by measuring that displaced by 5 M NaCl. The amount of chemokine displaced by CCL18 was then compared to the amount displaced by 5 M NaCl. As shown in Figure 8, convincing displacement was only achieved with the highest concentration of CCL18, but again the results demonstrated selectivity. The pattern observed correlated to some but not all interactions observed in the assay measuring displacement from the leukocytes. No displacement was observed for CCL5, suggesting in this case that the interaction between CCL5 and heparin is stronger than that between CCL18 and heparin. Whilst CXCL10 and CXCL11 displaced CCL18 from the surface of PBLs, CCL18 did not displace them from heparin, again demonstrating their strong interaction with this GAG. CCL2 and CCL4 did not displace CCL18, but they were displaced by CCL18, which is correlated by their relatively low affinity for heparin [36].

## Discussion

CCL18 has been reported to inhibit CCR3-mediated chemotactic responses [27] as well as to act as a modulator of CXCR4-dependent responses via the interaction with GPR30 [28]. These reports and the fact that CCL18 is constitutively present at high levels in human plasma, yet has a poor chemotactic activity, led us to further investigate its ability to inhibit other chemotactic responses mediated by homeostatic as well as inflammatory chemokines.

CCL18 abrogated CCR1, 2, 4 and 5 mediated chemotactic responses, whereas no effect was observed on chemotactic responses mediated by CCR7 and CCR9 or on CXCR3 and CXCR5 expressing cells. Thus CCL18 appears to inhibit chemokine receptors involved in inflammation, rather than those whose principal role is homeostasis.

The concentrations at which CCL18 exhibits its inhibitory activity are relatively high, but could be of physiological relevance since the concentration of the active chemokine compartment, believed to be that which is immobilized on cell surfaces, resulting in local high concentrations, is not measurable. Circulating CCL18, which would reflect the total amount, is constitutively present at high levels (20 ng/ml) but in disease is unregulated 4-fold [26]. In addition several human diseases have been reported to be accompanied with elevated levels of CCL18, in which the leukocyte recruitment was out of control (reviewed in 25). For example in synovial fluids from septic arthritis and rheumatoid arthritis patients levels of 140 ng/ml and 190 ng/ml CCL18 were determined, respectively, whereas in the non-inflammatory conditions of osteoarthritis and crystal induced arthritis, the levels of CCL18 are 34 and 38 ng/ml [37].

Certain chemokines have been reported to antagonise receptors other than their functional receptors. The CXCR3 ligands CXCL9, CXCL10 and CXCL11 were shown to act as antagonists for CCR3 [38], CCL11 was shown to be a natural antagonist for CCR2 [39] and CCL7 for CCR5 [40]. Thus these antagonistic effects of chemokines reflect a role for the fine-tuning of cellular responses mediated by an interplay of agonistic and antagonistic effects.

The antagonistic molecular mechanism of action of CCL18 on CCR3 based on its effect on functional responses of Ca^2+^ mobilization revealed that CCL18 acts as a surmountable antagonist showing no diminution in maximal response, demonstrating competitive inhibition. By definition an antagonist is termed competitive, when both agonist and antagonist compete for the same binding domain on the receptor, and thus bind in an orthosteric manner, and the relative affinity and concentrations of the agonist and antagonist determine which molecule occupies the binding site [41]. However as we used a functional assay in order to determine the molecular mechanism of action it is not possible to draw conclusions regarding the initial binding event. Equilibrium competition binding assays on L1.2/CCR3 transfectants suggested that CCL18 is not a pure competitive inhibitor, since CCL18 does not fully compete ^125^I-CCL11 binding.

Chemokine receptors have been reported to be found in different states, coupled, R*, and uncoupled, R [42]. For CXCR3, CXCL11 binds to both states whereas CXCL10 only binds to the R* form. Our results could be explained by the following hypothesis. CCL11 is able to bind to both receptor states, R and R*. However ^125^I-CCL18 only binds to one of these, as demonstrated by the considerably lower number of courts, only about 10% of those of ^125^I-CCL11, whereas both proteins had the same specific radioactivity. Since CCL18 is a competitive antagonist in the functional assay, it would appear that the same binding site for the two ligands is found on the R* state, hence CCL18 binds to this form. Whilst CCL18 is unable to totally displace bound CCL11, but is able to achieve complete inhibition of CCL11 mediated chemotaxis, is attributed to the fact that it binds to the activated R* state, which is the state that mediates functional effects. It is possible that a large proportion of the CCR3 receptors are found uncoupled in the L1.2 transfectants, since the number of receptors expressed may be far greater than the appropriate G proteins. Despite the fact that CCL18 binds to the R* state no activation of this receptor has been observed, either in induction of chemotaxis (data not shown) or in receptor internalization [33].

The studies of the interaction of CCL18 with CCR3 described here, as well as those previously reported, confirm that CCL18 interacts directly with the CCR3 receptor. Since CCL18 is able to inhibit other CC chemokine receptor induced responses, we investigated whether this effect was also due to a direct chemokine-receptor binding event. The inhibitory potencies could suggest a different mechanism since inhibition of CCR3 calcium mobilization and chemotaxis by CCL18 were in the nM range, whereas inhibition of the other receptors was predominantly µM. Analysis of fluorescent CCL18 binding to L1.2 parental cells, and to their transfectants revealed that specific receptor binding was only observed in CCR3 transfectants**.**

Anti-inflammatory properties mediated by CCL18 were further suggested by its ability to displace GAG-bound chemokines. The chemokine/GAG interaction has been proposed to present chemokines to their receptors in either a cis or trans mode [7]. Moreover the fact that chemokine binding to GAGs is necessary for receptor activation has previously been described, since removal of cell surface GAGs abrogates the ability of CCL5 to elicit Ca^2+^ mobilization [6]. We have shown that CCL18 displaces immobilised heparin bound chemokines, representing trans-presentation, which would *in vivo* lead to a disruption of the chemokine gradient and thus of the directional signal for the cells to migrate to the site of inflammation. We have also investigated heterologous displacement of CCL18 bound to cell surface GAGs, by using an equilibrium competition binding assay established with radiolabelled CCL18. Binding of CCL18 to the PBLs could be a combination of receptor binding as well as GAG binding, but since heparin almost totally displaces CCL18 from PBLs, and the ^44^AAGA^47^-CCL18 retains chemotactic activity equivalent to the parent chemokine (data not shown), but has very reduced binding capacity, this suggests that the binding observed is mainly due to GAG binding [33].

Cis-presentation on the leukocyte surface could serve two functions. The first is that this facilitates receptor binding by presenting the chemokine to the receptor. Alternatively, the close association of the proteoglycan to the receptor could act as a sink for the chemokine thereby suppressing receptor binding. The results obtained in this study suggest that CCL18 is able to reduce receptor activation by displacing the GAG bound chemokine, suggesting that for certain receptors cis-presentation is important and enhances activity. This would be the case for CCL11 and CCL26 activation of CCR3, CCL17 and CCL22 activation of CCR4 and CCL5 activation of CCR1 and/or CCR5, since these ligands displace CCL18 to a greater or lesser extent. On the contrary some receptors such as CCR7, CCR9, CXCR3 and CXCR5 activation appear to be independent of cis-presentation as heterologous displacement is observed, but receptor activation is not modulated by CCL18.

Competition with certain pairs suggested additional features. There was no displacement of ^125^I-CCL18 from PBL mediated by CCL4, to be expected since it only binds GAGs weakly, nor by CCL5, which is one of the chemokines that bind GAGs most strongly. This suggests that CCL5 and CCL18 bind to different GAGs on the cell surface demonstrating a differential GAG selectivity. On the other hand, the fact that CCL4 was considerably displaced by CCL18 corroborates their different binding capacity to heparin, whereas CCL5 was not displaced, again in concordance with its heparin binding properties. Displacement of CCL5 needs to be determined from a cell surface GAG such as heparan sulphate, or cultured cells which do not express CCL5 receptors, since heparin is not expressed on cell surfaces, but is found in the circulation, and was used in these preliminary experiments as a model GAG. Thus these results point to a more complex inter-relationship that has been identified to date, and remains to be more fully explored and elucidated. Extension to trans-presentation assays using endothelial cells such as HUVECs, will yield important information concerning the different classes of GAG that mediate binding to the leukocyte or those on the endothelial cell surface.

We have shown that the ^44^KRGR^47^ cluster of CCL18 plays a role in the interaction with CCR3, 4 and 5 since ^44^AAGA^47^-CCL18 shows decreased antagonistic activity. This could be attributed to the prevention of cis-presentation as described above, or to an overlapping epitope of the GAG and receptor binding sites as has previously been observed for GAG binding mutants of CCL5 and CCL3 which have overlapping epitopes for CCR1 and GAG binding [43,44].

Our results have suggested a regulatory or anti-inflammatory role of CCL18 by blocking the cellular recruitment mediated by several receptors as well as the displacement of GAG-bound chemokines, which might result in the disruption of the directional signal for cells to migrate. It remains to be elucidated whether CCL18 exhibits this role *in vivo* and under pathophysiological conditions associated with elevated levels of CCL18.

## References

[1] Zlotnik A, Yoshie O (2000) Chemokines: a new classification system and their role in immunity. Immunity 12: 121-127. doi:10.1016/S1074-7613(00)80165-X. PubMed: 10714678. 10714678 10.1016/s1074-7613(00)80165-x

[2] Bacon K, Baggiolini M, Broxmeyer H, Horuk R, Lindley I et al. (2002) Chemokine/chemokine receptor nomenclature. J Interferon Cytokine Res 22: 1067-1068. doi:10.1089/107999002760624305. PubMed: 12433287. 12433287 10.1089/107999002760624305

[3] Johnson Z, Proudfoot AE, Handel TM (2005) Interaction of chemokines and glycosaminoglycans: a new twist in the regulation of chemokine function with opportunities for therapeutic intervention. Cytokine Growth Factor Rev 16: 625-636. doi:10.1016/j.cytogfr.2005.04.006. PubMed: 15990353. 15990353 10.1016/j.cytogfr.2005.04.006

[4] Rot A (1992) Endothelial cell binding of NAP-1/IL-8: role in neutrophil emigration. Immunol Today 13: 291-294. doi:10.1016/0167-5699(92)90039-A. PubMed: 1510812. 1510812 10.1016/0167-5699(92)90039-A

[5] Proudfoot AEI, Handel TM, Johnson Z, Lau EK, LiWang P et al. (2003) Glycosaminoglycan binding and oligomerization are essential for the in vivo activity of certain chemokines. Proc Natl Acad Sci U S A 100: 1885-1890. doi:10.1073/pnas.0334864100. PubMed: 12571364. 12571364 10.1073/pnas.0334864100 PMC149928

[6] Burns JM, Gallo RC, DeVico AL, Lewis GK (1998) A new monoclonal antibody, mAb 4A12, identifies a role for the glycosaminoglycan (GAG) binding domain of RANTES in the antiviral effect against HIV-1 and intracellular Ca2+ signaling. J Exp Med 188: 1917-1927. doi:10.1084/jem.188.10.1917. PubMed: 9815269. 9815269 10.1084/jem.188.10.1917 PMC2212410

[7] Ali S, Hardy LA, Kirby JA (2003) Transplant immunobiology: a crucial role for heparan sulfate glycosaminoglycans? Transplantation 75: 1773-1782. doi:10.1097/01.TP.0000065805.97974.93. PubMed: 12811234. 12811234 10.1097/01.TP.0000065805.97974.93

[8] Hieshima K, Imai T, Baba M, Shoudai K, Ishizuka K et al. (1997) A novel human CC chemokine PARC that is most homologous to macrophage-inflammatory protein-1 alpha/LD78 alpha and chemotactic for T lymphocytes, but not for monocytes. J Immunol 159: 1140-1149. PubMed: 9233607. 9233607

[9] Wells TN, Peitsch MC (1997) The chemokine information source: identification and characterization of novel chemokines using the WorldWideWeb and expressed sequence tag databases. J Leukoc Biol 61: 545-550. PubMed: 9129202. 9129202 10.1002/jlb.61.5.545

[10] Li H, Ruben S Macrophage inflammatory protein-3 and -4. United States Patent Patent Number: 5,504,003. United States patent application

[11] Adema GJ, Hartgers F, Verstraten R, de Vries E, Marland G et al. (1997) A dendritic-cell-derived C-C chemokine that preferentially attracts naive T cells. Nature 387: 713-717. doi:10.1038/42716. PubMed: 9192897. 9192897 10.1038/42716

[12] Kodelja V, Müller C, Politz O, Hakij N, Orfanos CE et al. (1998) Alternative macrophage activation-associated CC-chemokine-1, a novel structural homologue of macrophage inflammatory protein-1 alpha with a Th2-associated expression pattern. J Immunol 160: 1411-1418. PubMed: 9570561. 9570561

[13] Schraufstatter I, Takamori H, Sikora L, Sriramarao P, Discipio RG (2004) Eosinophils and monocytes produce pulmonary and activation-regulated chemokine, which activates cultured monocytes/macrophages. Am J Physiol Lung Cell Mol Physiol 286: L494-L501. doi:10.1152/ajplung.00323.2002. PubMed: 12716654. 12716654 10.1152/ajplung.00323.2002

[14] Guan P, Burghes AH, Cunningham A, Lira P, Brissette WH et al. (1999) Genomic organization and biological characterization of the novel human CC chemokine DC-CK-1/PARC/MIP-4/SCYA18. Genomics 56: 296-302. doi:10.1006/geno.1998.5635. PubMed: 10087196. 10087196 10.1006/geno.1998.5635

[15] de Nadaï P, Charbonnier AS, Chenivesse C, Sénéchal S, Fournier C et al. (2006) Involvement of CCL18 in allergic asthma. J Immunol 176: 6286-6293. PubMed: 16670340. 16670340 10.4049/jimmunol.176.10.6286

[16] Günther C, Bello-Fernandez C, Kopp T, Kund J, Carballido-Perrig N et al. (2005) CCL18 is expressed in atopic dermatitis and mediates skin homing of human memory T cells. J Immunol 174: 1723-1728. PubMed: 15661937. 15661937 10.4049/jimmunol.174.3.1723

[17] Chenivesse C, Chang Y, Azzaoui I, Ait YS, Morales O et al. (2012) Pulmonary CCL18 recruits human regulatory T cells. J Immunol 189: 128-137. doi:10.4049/jimmunol.1003616. PubMed: 22649201. 22649201 10.4049/jimmunol.1003616

[18] Lindhout E, Vissers JL, Hartgers FC, Huijbens RJ, Scharenborg NM et al. (2001) The dendritic cell-specific CC-chemokine DC-CK1 is expressed by germinal center dendritic cells and attracts CD38-negative mantle zone B lymphocytes. J Immunol 166: 3284-3289. PubMed: 11207283. 11207283 10.4049/jimmunol.166.5.3284

[19] Schutyser E, Struyf S, Proost P, Opdenakker G, Laureys G et al. (2002) Identification of biologically active chemokine isoforms from ascitic fluid and elevated levels of CCL18/pulmonary and activation-regulated chemokine in ovarian carcinoma. J Biol Chem 277: 24584-24593. doi:10.1074/jbc.M112275200. PubMed: 11978786. 11978786 10.1074/jbc.M112275200

[20] Vulcano M, Struyf S, Scapini P, Cassatella M, Bernasconi S et al. (2003) Unique regulation of CCL18 production by maturing dendritic cells. J Immunol 170: 3843-3849. PubMed: 12646652. 12646652 10.4049/jimmunol.170.7.3843

[21] Atamas SP, Luzina IG, Choi J, Tsymbalyuk N, Carbonetti NH et al. (2003) Pulmonary and activation-regulated chemokine stimulates collagen production in lung fibroblasts. Am J Respir Cell Mol Biol 29: 743-749. doi:10.1165/rcmb.2003-0078OC. PubMed: 12805086. 12805086 10.1165/rcmb.2003-0078OC

[22] Prasse A, Pechkovsky DV, Toews GB, Jungraithmayr W, Kollert F et al. (2006) A vicious circle of alveolar macrophages and fibroblasts perpetuates pulmonary fibrosis via CCL18. Am J Respir Crit Care Med 173: 781-792. doi:10.1164/rccm.200509-1518OC. PubMed: 16415274. 16415274 10.1164/rccm.200509-1518OC

[23] Schraufstatter IU, Zhao M, Khaldoyanidi SK, Discipio RG (2012) The chemokine CCL18 causes maturation of cultured monocytes to macrophages in the M2 spectrum. Immunology 135: 287-298. doi:10.1111/j.1365-2567.2011.03541.x. PubMed: 22117697. 22117697 10.1111/j.1365-2567.2011.03541.x PMC3372745

[24] Chang Y, de Nadai P, Azzaoui I, Morales O, Delhem N et al. (2010) The chemokine CCL18 generates adaptive regulatory T cells from memory CD4+ T cells of healthy but not allergic subjects. FASEB J 24: 5063-5072. doi:10.1096/fj.10-162560. PubMed: 20702776. 20702776 10.1096/fj.10-162560

[25] Schutyser E, Richmond A, Van Damme J (2005) Involvement of CC chemokine ligand 18 (CCL18) in normal and pathological processes. J Leukoc Biol 78: 14-26. doi:10.1189/jlb.1204712. PubMed: 15784687. 15784687 10.1189/jlb.1204712 PMC2665283

[26] Struyf S, Schutyser E, Gouwy M, Gijsbers K, Proost P et al. (2003) PARC/CCL18 is a plasma CC chemokine with increased levels in childhood acute lymphoblastic leukemia. Am J Pathol 163: 2065-2075. doi:10.1016/S0002-9440(10)63564-X. PubMed: 14578205. 14578205 10.1016/S0002-9440(10)63564-X PMC1892433

[27] Nibbs RJ, Salcedo TW, Campbell JD, Yao XT, Li Y et al. (2000) C-C chemokine receptor 3 antagonism by the beta-chemokine macrophage inflammatory protein 4, a property strongly enhanced by an amino-terminal alanine-methionine swap. J Immunol 164: 1488-1497. PubMed: 10640766. 10640766 10.4049/jimmunol.164.3.1488

[28] Catusse J, Wollner S, Leick M, Schröttner P, Schraufstätter I et al. (2010) Attenuation of CXCR4 responses by CCL18 in acute lymphocytic leukemia B cells. J Cell Physiol 225: 792-800. doi:10.1002/jcp.22284. PubMed: 20568229. 20568229 10.1002/jcp.22284

[29] Chen J, Yao Y, Gong C, Yu F, Su S et al. (2011) CCL18 from tumor-associated macrophages promotes breast cancer metastasis via PITPNM3. Cancer Cell 19: 541-555. doi:10.1016/j.ccr.2011.02.006. PubMed: 21481794. 21481794 10.1016/j.ccr.2011.02.006 PMC3107500

[30] Proudfoot AE, Borlat F (2000) Purification of recombinant chemokines from E. coli. Methods Mol Biol 138: 75-87. PubMed: 10840744. 10840744 10.1385/1-59259-058-6:75

[31] Severin IC, Souza AL, Davis JH, Musolino N, Mack M et al. (2012) Properties of 7ND-CCL2 are modulated upon fusion to Fc. Protein Eng Des Sel 25: 213-222. doi:10.1093/protein/gzs008. PubMed: 22388887. 22388887 10.1093/protein/gzs008

[32] Severin IC, Gaudry JP, Johnson Z, Kungl A, Jansma A et al. (2010) Characterization of the chemokine CXCL11-heparin interaction suggests two different affinities for glycosaminoglycans. J Biol Chem 285: 17713-17724. doi:10.1074/jbc.M109.082552. PubMed: 20363748. 20363748 10.1074/jbc.M109.082552 PMC2878535

[33] Krohn S, Garin A, Gabay C, Proudfoot AEI (2013) The activity of CCL18 is principally mediated through interaction with glycosaminoglycans. Frontiers in Immunology/ Chemoattractants in *press* . 10.3389/fimmu.2013.00193 PMC3711072 23874339

[34] Arunlakshana O, Schild HO (1959) Some quantitative uses of drug antagonists. PMC1481829: 48-58. 10.1111/j.1476-5381.1959.tb00928.x PMC1481829 13651579

[35] Schild HO (1949) pAx and competitive drug antagonism. Br J Pharmacol 4: 277-280. PubMed: 18141089. 10.1111/j.1476-5381.1949.tb00548.x PMC1509912 18141089

[36] Kuschert GS, Coulin F, Power CA, Proudfoot AE, Hubbard RE et al. (1999) Glycosaminoglycans interact selectively with chemokines and modulate receptor binding and cellular responses. Biochemistry 38: 12959-12968. doi:10.1021/bi990711d. PubMed: 10504268. 10504268 10.1021/bi990711d

[37] Schutyser E, Struyf S, Wuyts A, Put W, Geboes K et al. (2001) Selective induction of CCL18/PARC by staphylococcal enterotoxins in mononuclear cells and enhanced levels in septic and rheumatoid arthritis. Eur J Immunol 31: 3755-3762. doi:10.1002/1521-4141(200112)31:12. PubMed: 11745396. 11745396 10.1002/1521-4141(200112)31:12<3755::aid-immu3755>3.0.co;2-o

[38] Loetscher P, Pellegrino A, Gong JH, Mattioli I, Loetscher M et al. (2001) The ligands of CXC chemokine receptor 3, I-TAC, Mig, and IP10, are natural antagonists for CCR3. J Biol Chem 276: 2986-2991. doi:10.1074/jbc.M005652200. PubMed: 11110785. 11110785 10.1074/jbc.M005652200

[39] Ogilvie P, Bardi G, Clark-Lewis I, Baggiolini M, Uguccioni M (2001) Eotaxin is a natural antagonist for CCR2 and an agonist for CCR5. Blood 97: 1920-1924. doi:10.1182/blood.V97.7.1920. PubMed: 11264152. 11264152 10.1182/blood.v97.7.1920

[40] Blanpain C, Migeotte I, Lee B, Vakili J, Doranz BJ et al. (1999) CCR5 binds multiple CC-chemokines: MCP-3 acts as a natural antagonist. Blood 94: 1899-1905. PubMed: 10477718. 10477718

[41] Kenakin P (1900) A Pharmacology Primer-orthosteric drug antagonism. In: A Pharmacology Primer, 3 edition: theory, applications and methods

[42] Cox MA, Jenh CH, Gonsiorek W, Fine J, Narula SK et al. (2001) Human interferon-inducible 10-kDa protein and human interferon-inducible T cell alpha chemoattractant are allotopic ligands for human CXCR3: differential binding to receptor states. Mol Pharmacol 59: 707-715. PubMed: 11259614. 11259614 10.1124/mol.59.4.707

[43] Proudfoot AE, Fritchley S, Borlat F, Shaw JP, Vilbois F et al. (2001) The BBXB motif of RANTES is the principal site for heparin binding and controls receptor selectivity. J Biol Chem 276: 10620-10626. doi:10.1074/jbc.M010867200. PubMed: 11116158. 11116158 10.1074/jbc.M010867200

[44] Graham GJ, Wilkinson PC, Nibbs RJ, Lowe S, Kolset SO et al. (1996) Uncoupling of stem cell inhibition from monocyte chemoattraction in MIP-1alpha by mutagenesis of the proteoglycan binding site. EMBO J 15: 6506-6515. PubMed: 8978677. 8978677 PMC452475

